# Roles and Therapeutic Targeting of Exosomes in Sepsis‐Induced Cardiomyopathy

**DOI:** 10.1111/jcmm.70559

**Published:** 2025-04-23

**Authors:** Rui Fan, Han Liu, Qun Liang

**Affiliations:** ^1^ Graduate School Heilongjiang University of Chinese Medicine Harbin China; ^2^ Graduate School University College London London UK; ^3^ Department of Critical Care Medicine First Affiliated Hospital of Heilongjiang University of Chinese Medicine Harbin China

**Keywords:** exosome, inflammation, mesenchymal stem cell, myocardial injury, sepsis‐induced cardiomyopathy

## Abstract

Sepsis‐induced cardiomyopathy (SICM) is a complex and fatal manifestation of sepsis, characterised by myocardial dysfunction that exacerbates the clinical prognosis in septic patients. While the pathophysiology of SICM remains incompletely understood, emerging evidence highlights the multifaceted functions of exosomes, small membrane‐bound extracellular vesicles, in mediating the inflammatory responses and cardiac dysfunction involved in this condition. During sepsis, exosomes are secreted by various cells, such as cardiomyocytes, endothelial cells and macrophages, which serve as critical messengers, transferring proteins, lipids and RNA molecules that influence recipient cells, thus affecting cellular functions and disease progression. This review summarises the pathophysiology of SICM and the basics of exosomes and focuses on exosome‐mediated mechanisms in SICM, including their role in inflammation, oxidative stress, mitochondrial dysfunction and myocardial injury, offering novel insights into the exosome‐based therapeutic strategies in SICM.

**Abbreviations**
ATF2: activating transcription factor 2
circRNA: circular RNA
DRP1: dynamin‐related protein 1
ESCRT: endosomal sorting complex required for transport
GSDM: gasdermin
GSH: glutathione
HMBOX1: homeobox containing 1
HSPA12B: heat shock protein A12B
I/R: Ischemia/reperfusion
IGF‐1: insulin‐like growth factor 1
IL‐1β: interleukin‐1beta
ILVs: intraluminal vesicles
lncRNA: long non‐coding RNA
LPS: lipopolysaccharide
MAP K4: mitogen‐activated protein kinase 4
miRNA: microRNA
MSCs: mesenchymal stem cells
MVBs: multivesicular bodies
NET: neutrophil extracellular trap
NLRP3: NOD‐like receptor family pyrin domain containing 3
NO: nitric oxide
PGE2: prostaglandin E2
PINK1: PTEN‐induced putative kinase 1
ROS: reactive oxygen species
SEMA3A: semaphorin 3a
SICM: sepsis‐induced cardiomyopathy
SLC2A1: solute carrier family 2 member 1
SOD: superoxide dismutase
STAT3: signal transducer and activator of transcription 3
TLRs: Toll‐like receptors
TNFSF10: tumour necrosis factor superfamily member 10
TNF‐α: tumour necrosis factor‐alpha
TXNIP: thioredoxin‐interacting protein

## 1 Introduction

Sepsis‐induced cardiomyopathy (SICM) is a severe complication of sepsis, characterised by impaired myocardial contractility, altered haemodynamics and even cardiogenic shock, which contributes to the high morbidity and mortality in septic patients [1, 2]. Despite extensive research, the pathophysiological mechanisms underlying SICM remain incompletely understood, with no specific therapeutic strategies yet available to mitigate or reverse the cardiac dysfunction associated with sepsis [3, 4]. Recent insights into the role of exosomes have identified that they function as key mediators of cellular communication in SICM. Exosomes are small, membrane‐bound vesicles with a diameter of 30–150 nm are secreted by virtually all cell types and carry a diverse array of bioactive molecules, including proteins, lipids, RNAs like mRNA and non‐coding RNAs and other molecular cargo [5, 6]. These vesicles facilitate the transfer of information between cells and organs, influencing a broad range of physiological processes such as immune response, inflammation, tissue repair and cellular apoptosis [7, 8]. Importantly, during SICM progression, exosomes have been implicated in various pathophysiological processes, such as inflammation, myocardial injury and cardiac dysfunction [9, 10].

Exosomes mediate the communication among cardiomyocytes, endothelial cells, and macrophages during SICM. Exosomes derived from lipopolysaccharide (LPS)‐induced cardiomyocytes exhibit decreased protein expression that is associated with exosomal biogenesis, participating in enhanced cardiac cell death [11]. Simultaneously, exosomes secreted by endothelial cells influence local cardiac responses and modulate the apoptosis‐related pathway, thereby reducing myocardial injury during sepsis [12]. Moreover, exosomes derived from LPS‐stimulated macrophages promote the release of pro‐inflammatory cytokines and trigger cardiac inflammation and myocardial depression, indicating the detrimental role of macrophage‐derived exosomes in SICM [13]. Given their role in disease progression, exosomes have gained attention as novel therapeutic targets in this disease. Mesenchymal stem cells (MSCs) are multipotent stromal cells capable of differentiating into various cell types, including osteocytes, chondrocytes and adipocytes [14]. MSC‐derived exosomes are critical in mediating the therapeutic effects of MSCs, which play a crucial role in cell‐to‐cell communication by transferring bioactive molecules to recipient cells, influencing their behaviour and function [15, 16]. Exosomes from MSCs have been demonstrated to exert anti‐inflammatory and anti‐apoptotic effects in SICM, which alleviate myocardial function and inflammation in patients with sepsis‐induced myocardial injury [17, 18].

This review concisely discusses the pathophysiology of SICM and the basics of exosomes, as well as emphasises the interplay between exosomes and various origins, including circulating blood, cardiomyocytes, endothelial cells, macrophages and platelets during SICM progression. Additionally, it explores the therapeutic potential of exosomes derived from MSCs, identifying critical knowledge gaps and proposing directions for future research that may facilitate the translation of exosome‐based strategies into clinical practice for patients with SICM.

## 2 Pathophysiology of SICM

**FIGURE 1:**
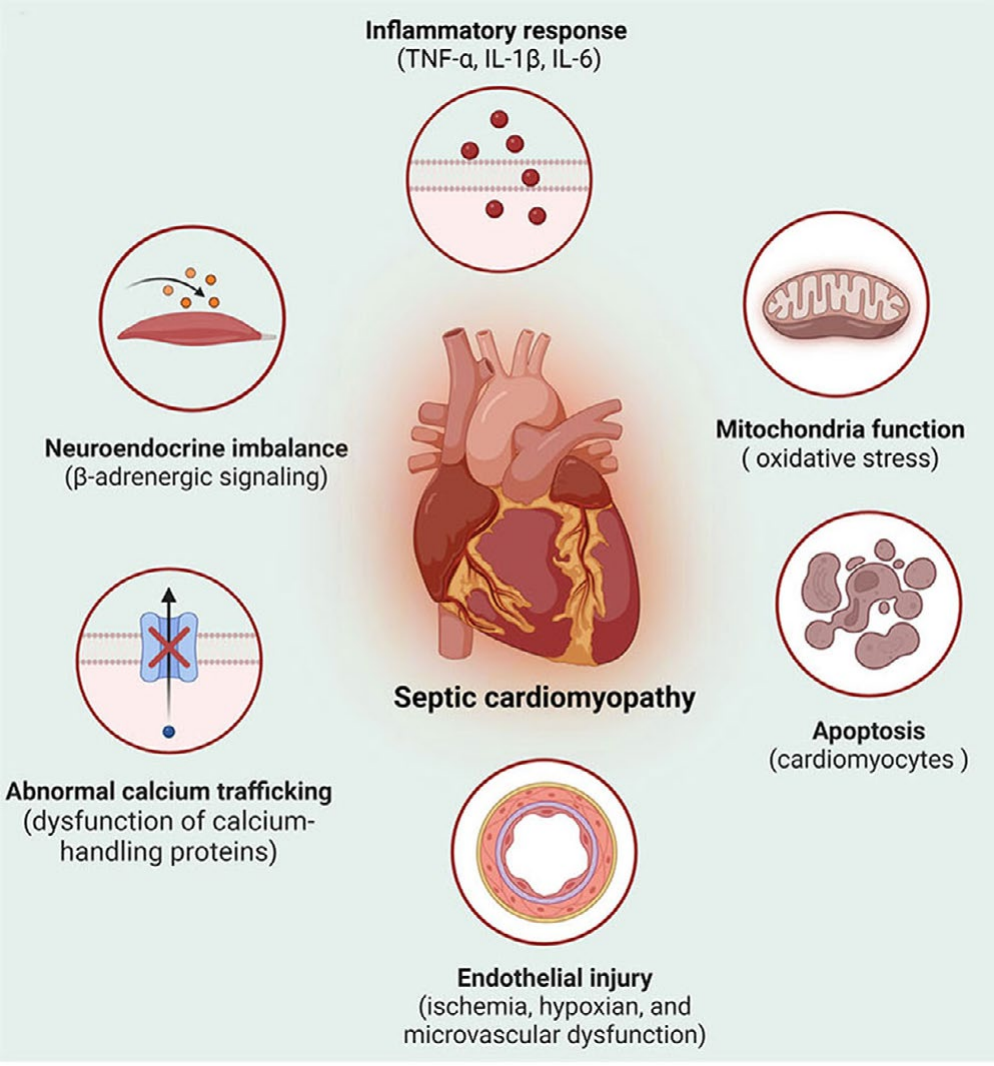
The pathophysiology of sepsis‐induced cardiomyopathy. Various biological processes are involved in sepsis‐induced cardiomyopathy, including the release of inflammatory mediators, cardiomyocyte apoptosis, mitochondrial dysfunction, endothelial injury, neuroendocrine disturbance and abnormalities in calcium handling.

The pathogenic mechanisms of SICM are intricate, involving a confluence of cellular, molecular and hemodynamic changes that culminate in impaired myocardial performance [19]. As the key elements driving SICM, several pathophysiological processes, including inflammatory responses, myocardial apoptosis, mitochondrial dysfunction, contractile impairment, endothelial injury and neuroendocrine imbalance, are considered to cause impairment of cardiac function in septic patients (Figure 1).

The initial insult in sepsis is typically a microbial infection, whose pathogen‐associated molecular patterns, such as LPS and lipoteichoic acid, contribute to the activation of pattern recognition receptors such as Toll‐like receptors (TLRs) on immune cells [20, 21]. This triggers the activation of NF‐κB signalling pathways, leading to the increased expression of pro‐inflammatory cytokines, such as tumour necrosis factor‐alpha (TNF‐α), interleukin‐1beta (IL‐1β) and IL‐6 [22]. These inflammatory factors can initiate the extrinsic apoptotic pathway through the activation of death receptors on the surface of cardiomyocytes and further the activation of caspase enzymes, which in turn dismantle the cellular machinery, leading to cell apoptosis [23]. TNF‐α can induce the overproduction of reactive oxygen species (ROS) and exacerbates oxidative stress in the myocardium, contributing to decreased mitochondrial membrane potential and impaired ATP synthesis, ultimately resulting in mitochondrial dysfunction [24, 25]. Cardiomyocyte apoptosis and mitochondrial dysfunction impair myocardial integrity and function, thereby leading to compromised myocardial contractility, which is characterised by a reduced left ventricular ejection fraction and impaired systolic and diastolic function, despite the presence of adequate preload and increased cardiac output [26]. Besides, in response to inflammatory cytokines and oxidative stress, downregulation of contractile proteins in cardiomyocytes, such as myosin heavy chain and actin, aggravates myocardial dysfunction and contractile impairment [27]. During sepsis, inflammatory responses alter the expression and function of calcium‐handling proteins in myocardial cells, such as the sarcoplasmic reticulum calcium ATPase, ryanodine receptors and sodium‐calcium exchangers, and disrupt the balance between calcium influx and efflux, thus causing a decrease in contractile force and overall cardiac output [28].

Endothelial dysfunction also plays a pivotal role in the pathogenesis of SICM by impairing myocardial perfusion. In sepsis, prolonged inflammatory responses affect the endothelial cells lining the blood vessels in the coronary circulation. Cytokines, such as TNF‐α, IL‐1β and IL‐6, along with other mediators like nitric oxide (NO), result in an imbalance between vasodilation and vasoconstriction [29, 30]. Other factors such as complement activation and increased expression of cell adhesion molecules lead to a state of microvascular vasodilation, causing a disturbance between oxygen supply and demand that further exacerbates myocardial ischemia and hypoxia [31, 32]. Microvascular dysfunction is accompanied by increased vascular permeability, which leads to edema and interstitial fluid accumulation in the myocardium, further hindering oxygen and nutrient delivery to cardiomyocytes [33]. This creates a vicious cycle of tissue hypoxia and damage, which elicits both systolic and diastolic dysfunction in the septic heart. Moreover, sepsis can induce dysfunction of the hypothalamic–pituitary–adrenal axis, leading to autonomic nervous system dysfunction and decreased myocardial contractility. This neuroendocrine imbalance can further impair cardiac function by reducing the myocardium's responsiveness to sympathetic neurotransmitters [34]. Also, NO and ROS can depress myocardial function by downregulating the β‐adrenergic signalling pathways [35].

The pathophysiology of SICM is complex and multifaceted, involving a combination of inflammatory responses, impaired myocardial contractility, endothelial dysfunction and neuroendocrine disturbance. Further research studies on these pathogenic mechanisms are crucial for improving treatment strategies since current therapies targeting a single pathway have not been effective. Shedding light on the role of exosomes in SICM may offer new perspectives on the molecular mechanisms underlying this condition.

## 3 Overviews of Exosomes

### 3.1 Biogenesis

**FIGURE 2:**
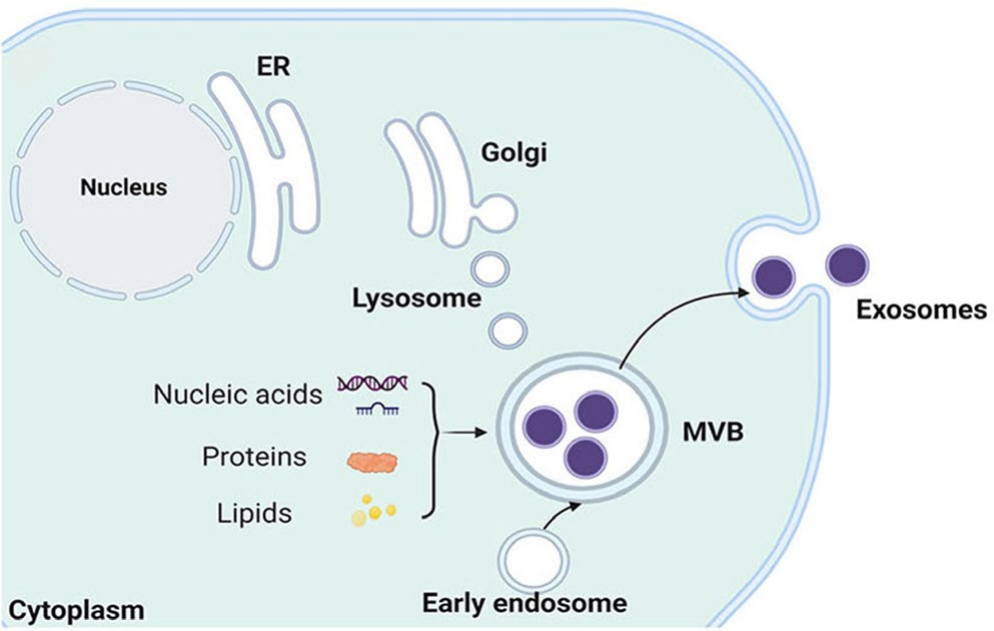
Biogenesis of exosomes. Early endosomes are formed by endocytosis of the parent cell. They then undergo the second invagination of the plasma membrane, thus forming ILVs, and the endosomes that enclose the ILVs are MVBs. MVBs can fuse with the plasma membrane and release the ILVs, namely exosomes, which contain various components, such as nucleic acids, proteins and lipids. ER, endoplasmic reticulum; ILVs, intraluminal vesicles; MVBs, multivesicular bodies.

Exosomes are small, membranous vesicles, typically ranging from 30 to 150 nm in diameter, that are secreted by most cell types into the extracellular space [6]. They play pivotal roles in intercellular communication through the transfer of bioactive molecules such as proteins, lipids and nucleic acids. The biogenesis of exosomes is an intricate process involving multiple cellular pathways and molecular mechanisms (Figure 2). Exosome biogenesis begins with the formation of intracellular multivesicular bodies (MVBs) and culminates in the fusion of MVBs with the plasma membrane, facilitating the release of exosomes into the extracellular milieu. Endocytosis of cell surface molecules and the internalisation of extracellular material trigger the formation of early endosomes, which subsequently mature into late endosomes, whose membrane further invaginates to form internal vesicles within the lumen, referred to as intraluminal vesicles (ILVs) that are regarded as the precursors of exosomes [36, 37]. This process is regulated by several molecular mechanisms, including the endosomal sorting complex required for transport (ESCRT) machinery and ESCRT‐independent pathways. The ESCRT complexes recognise ubiquitinated proteins to promote vesicle scission from the membrane, thereby leading to the formation of ILVs within the late endosome; moreover, the ESCRT‐III complex and ALG‐2‐interacting protein X are responsible for the membrane pinching off to release ILVs into the lumen in the final scission step [38, 39]. Alternatively, ESCRT‐independent pathways, such as tetraspanins CD63, CD81 and CD9, which act as scaffolding proteins, organise lipid microdomains that are implicated in the budding of ILVs; also, the endosomal membrane lipid composition like ceramide, sphingomyelin and phosphatidylserine can influence ILV formation through the generation of membrane curvature that facilitates vesicle budding [40, 41]. During the maturation of late endosomes into MVBs, the sorting and selective packaging of cargo into ILVs occur, which ensures that specific proteins, lipids and nucleic acids are concentrated into the vesicles for eventual delivery to recipient cells [42]. The composition of exosomal cargo is dynamic and cell‐type specific, depending on various signals and the cellular context. The final step in exosome biogenesis is the fusion of MVBs with the plasma membrane, which allows the release of ILVs into the extracellular environment as exosomes. Several key proteins, such as Rab GTPases and soluble N‐ethylmaleimide‐sensitive factor attachment receptor proteins, regulate vesicle tethering, docking and fusion and mediate membrane fusion by forming complexes that bring the vesicular and target membranes into close proximity, facilitating the release of exosomes [43, 44].

### 3.2 Cargo Composition and Function

The typical cargo composition of exosomes contains proteins, lipids, nucleic acids and other biomolecules, which serve as vehicles for intercellular communication, influencing diverse physiological and pathological processes [45, 46]. The cargo composition of exosomes is dynamic and reflects the cellular environment and the functional state of the donor cell. Exosomes are rich in proteins, which include membrane proteins and cytosolic proteins that are involved in vesicle formation, trafficking and intercellular communication [47]. Exosomal membranes contain various proteins, including receptors, adhesion molecules and transporters, which not only reflect the cellular origin of the exosomes but also allow exosomes to mediate specific cellular interactions upon fusion with recipient cells [48]. In addition, exosomes contain various signalling proteins, including kinases, phosphatases and proteins involved in cellular stress responses, by which exosomes transfer signalling information to affect the behaviour of recipient cells [49]. Besides, exosomes can carry nucleic acids, including mRNA and non‐coding RNA, such as microRNA (miRNA), long non‐coding RNA (lncRNA) and circular RNA (circRNA), all of which can be transferred to recipient cells, influencing gene expression and cellular functions [50, 51, 52]. Exosomal mRNAs can be translated into proteins upon delivery to recipient cells, influencing cellular functions [53]. Non‐coding RNAs in exosomes function in several ways, such as modulating gene expression at the transcriptional level and interacting with chromatin, thereby regulating a variety of biological processes, including cell proliferation, apoptosis, differentiation, and immune regulation [54]. Additionally, the lipid composition of exosomes is crucial for their structural integrity and function. Exosomal lipids can influence membrane organisation and biological activity, and they also act as signalling molecules to modulate metabolic pathways of recipient cells [55, 56].

### 3.3 Exosome Isolation, Characterisation, and Storage

Exosome‐based research has gained significant attention in recent years due to their potential as biomarkers and therapeutic targets. The successful application of exosomes in both research and clinical settings heavily relies on efficient isolation, accurate characterisation and proper storage techniques. The first and most critical step in exosome‐based studies is their isolation from biological fluids. Ultracentrifugation remains the gold standard for exosome isolation due to its ability to efficiently isolate exosomes from complex biological matrices like plasma, serum and urine. It relies on the differential centrifugation of the sample at high speeds, allowing the precipitation of exosomes based on their size and density [57]. The process is time‐consuming, requires a high‐speed centrifuge and leads to the co‐isolation of non‐exosomal particles, such as apoptotic bodies or microvesicles, resulting in low purity. Additionally, the method is not scalable for large‐volume isolation, which may limit its application in high‐throughput or clinical settings [58]. Size‐exclusion chromatography (SEC) has emerged as a promising alternative to ultracentrifugation, offering high purity and preservation of exosomal integrity. This technique separates particles based on their size, effectively isolating exosomes while removing contaminants like proteins and lipoproteins. Thus, it has the advantage of being relatively faster and less labour‐intensive compared to ultracentrifugation, making it a suitable choice for clinical applications where sample turnaround time is critical [59]. SEC has limitations in terms of throughput and scalability, especially when large volumes of biological fluid are required. Additionally, the resolution of SEC may not be as high as ultracentrifugation in separating very small exosomes from other nanoparticles, potentially resulting in some contamination [60]. Immunoaffinity‐based methods, such as immunomagnetic bead‐based isolation, utilise antibodies that target exosome surface markers to selectively capture exosomes. These methods allow for high specificity and purity, which is crucial when studying exosomes from particular cell types or disease states. Immunoaffinity capture can also be highly quantitative, providing a better yield of exosomes from small sample volumes [61]. It relies on surface markers, which may not be universally expressed across all exosomes, leading to incomplete isolation of exosomes from certain sources. Furthermore, the isolation may be biased toward specific subpopulations of exosomes, potentially overlooking important vesicles that lack the targeted markers [62]. Precipitation methods, such as those utilising polyethylene glycol or commercial exosome isolation kits, are simple, cost‐effective and require minimal specialised equipment. The main limitation of precipitation methods is their lack of specificity, leading to contamination from non‐vesicular components such as proteins, lipoproteins and cellular debris. This can affect the purity and yield of isolated exosomes and may introduce variability into downstream analyses [63].

After isolation, accurate characterisation of exosomes is essential to ensure their identity and functionality. Nanoparticle tracking analysis (NTA) is a widely used method for exosome characterisation, as it allows for the measurement of exosome size distribution and concentration in real time. By tracking the Brownian motion of exosomes, NTA provides high‐resolution data regarding particle size and concentration, which are critical for understanding exosome populations in various biological fluids [64]. NTA is limited in its ability to differentiate between exosomes and other particles of similar size, such as apoptotic bodies and microvesicles. It can be sensitive to sample heterogeneity, and the presence of aggregates or debris can skew the results [65]. Transmission electron microscopy (TEM) is the gold standard for visualising exosomes at the ultrastructural level. It provides direct imaging of the vesicular morphology, allowing for the confirmation of exosome size and shape, typically ranging from 30 to 150 nm. TEM also provides detailed insights into the integrity of the exosomal membrane [66]. However, TEM is labour‐intensive, requires high expertise and is not suitable for high‐throughput analysis. The sample preparation process, including fixation and staining, may alter the exosome structure, making it difficult to preserve their natural morphology [67]. Western blotting is widely used to confirm the presence of exosome‐specific markers and to assess the purity of the isolated exosomes. This method is highly sensitive and provides valuable information regarding the protein content of exosomes, but it requires a known antibody to target a specific exosomal marker, limiting its ability to characterise exosomes from novel sources or those with uncharacterised markers [68].

Generally, exosomes are stored at −80°C or in liquid nitrogen. Freezing exosomes at low temperatures preserves their structural and molecular integrity, including protein and RNA content, for extended periods. This is essential for maintaining reproducibility across experiments, especially in long‐term studies [69]. However, the freeze–thaw process can lead to the aggregation or degradation of exosomes, particularly if they are not stored properly or if cycles of freezing and thawing are repeated. Moreover, the freezing process itself may alter the exosomal membrane, potentially affecting downstream analyses such as RNA profiling or protein assays [70].

### 3.4 Characteristics of Circulating Exosomes

Plasma is one of the most commonly used sources for exosome isolation in sepsis research due to its ease of collection and its rich content of vesicles derived from multiple organs and cell types. Plasma exosomes reflect a broad spectrum of physiological and pathological processes, including inflammation, endothelial dysfunction and myocardial injury, making them particularly relevant for studying SICM [71]. Additionally, plasma is already part of the standard diagnostic workup in clinical settings, which facilitates its use for biomarker discovery and clinical diagnostics [72]. However, the presence of large amounts of proteins, lipoproteins and other contaminants in plasma can complicate the purification of exosomes. Furthermore, while plasma exosomes reflect systemic inflammation, their potential to provide organ‐specific information, especially for cardiac injury, is limited due to the dilution effect and the shared nature of the exosome pool across different organs [73]. Serum, unlike plasma, is devoid of clotting factors, which makes serum a desirable alternative for exosome analysis, particularly in clinical settings where clotting factors are a concern. Serum exosomes are considered to reflect the biological and pathological state of the individual and can carry valuable biomarkers for SICM, including specific miRNAs and proteins linked to cardiac injury and inflammation [74]. Serum is also widely used in clinical diagnostics, providing a well‐established and minimally invasive option for exosome‐based research and biomarker development. However, serum collection involves blood clotting, which could lead to the release of cellular contents, including cellular fragments that might contaminate the exosome preparations, which impacts the purity and reproducibility of results [75]. Additionally, similar to plasma, serum exosomes may not provide specific insights into cardiac injury due to the systemic nature of the samples, limiting their ability to identify localised organ damage [76]. Urine exosomes could be an attractive alternative in the study of SICM due to their potential for reflecting kidney‐related pathophysiological changes associated with sepsis, such as acute kidney injury, which frequently co‐occurs with SICM. Urine exosomes are relatively easy to collect and contain both organ‐specific and systemic markers that can be indicative of sepsis severity and prognosis [77]. Importantly, urine‐derived exosomes offer the possibility of non‐invasive, real‐time monitoring of the disease state, which could be particularly beneficial in clinical settings [78]. One of the main challenges is the relatively low concentration of exosomes in urine, which requires highly sensitive techniques for isolation and characterisation. Additionally, contamination from urinary proteins and debris, as well as variation in urine composition based on hydration levels or other physiological factors, can introduce variability in results [79].

## 4 Role of Exosomes Derived From Various Origins in SICM

Exosomes, derived from circulating blood and various cell types, can influence cardiac function during SICM progression (Table 1). They exert dual functions in sepsis, as they can either exacerbate myocardial injury or offer protective effects, depending on their origin and molecular content.

**TABLE 1:** The role of exosomes in sepsis‐induced cardiomyopathy.

Exosomal components	Origin	Targets	Recipient cells	Effects	Ref.
ROS	Circulating blood	Undefine	Endothelial cells	Promote endothelial hyperpermeability and cardiac dysfunction	[80]
miR‐885‐5p	Circulating blood	HMBOX1	Cardiomyocytes	Induce cardiomyocyte pyroptosis	[81]
hsa‐miR‐1262	Circulating blood	SLC2A1	Cardiomyocytes	Promote apoptosis in myocardial cells	[82]
miRNAs	Cardiomyocytes	Pro‐angiogenic genes	Endothelial cells	Facilitate cell proliferation and angiogenesis	[83]
lncRNA KLF3‐AS1	Cardiomyocytes	miR‐23c/STAT5B	Cardiomyocytes	Enhance cardiomyocyte viability and alleviate myocardial injury	[84]
HSPA12B	Endothelial cells	NF‐κB	Macrophages	Decrease inflammatory responses and cardiac injury	[85]
miRNAs	Endothelial cells	BAK1, P53 and PTEN	Cardiomyocytes	Promote cell survival and reduce myocardial injury	[12]
Undefine	Endothelial cells	PI3K/AKT and NF‐κB	Undefine	Ameliorate inflammation, cardiomyocyte dysfunction, and myocardial injury	[86]
Undefine	Macrophages	Undefine	Undefine	Reduce cardiac inflammation and myocardial depression	[13]
TXNIP‐NLRP3	Monocytes	Caspase‐1, IL‐1β and IL‐18	Macrophages	Exacerbating cardiovascular inflammation	[87]
lncRNA Snhg14	M1 macrophage	miR‐181a‐5p/HMGB1/NF‐κB	Cardiomyocytes	Mitigate cardiac damage	[88]
miR‐146a	Macrophages	MAPK4/DRP1	Undefine	Inhibit inflammatory responses and improve mitochondrial function	[89]
miR‐24‐3p	M2 macrophage	TNFSF10	Cardiomyocytes	Reduce cardiomyocyte apoptosis and improve cardiac function	[90]
Superoxide	Platelets	Undefine	Vascular cells	Induce apoptosis in vascular cells and lead to inotropic dysfunction in the heart	[91]
Undefine	Platelets	Caspase‐3	Endothelial cells	Contribute to septic vascular dysfunction	[92]
HMGB1, miR‐15b‐5p and miR‐378a‐3p	Platelets	AKT/mTOR	Neutrophil	Promote the formation of neutrophil extracellular trap	[93]

### 4.1 Circulating Blood

The interaction between blood‐borne factors and cardiac tissue is central to the development of SICM, involving inflammatory mediators and oxidative stress that contribute to cardiac dysfunction [94]. It is reported that elevated levels of plasma exosomes are related to the severity of organ failure and predictive of mortality in critically ill patients with sepsis [95]. Exosomes derived from circulating blood during sepsis play a crucial role in mediating cardiomyopathy. For example, circulating exosomes collected from septic mice are enriched with high amounts of ROS, which is transported to endothelial cells, resulting in the production of podosome, an actin‐based dynamic membrane structure that is responsible for extracellular matrix degradation and angiogenesis, thereby causing endothelial hyperpermeability and cardiac dysfunction through fragmentation of zonula occludens‐1 [80]. Additionally, exosomes from the blood of septic patients have been shown to promote cardiomyocyte pyroptosis, a form of programmed cell death associated with inflammation. Septic rats treated with these exosomes exhibit high levels of serum inflammatory cytokines like IL‐1β and IL‐18, which are associated with increased pyroptosis‐related proteins in hearts. Further mechanistic evaluation revealed that miR‐885‐5p is upregulated to inhibit the expression of homeobox containing 1 (HMBOX1), thus leading to overexpression of NOD‐like receptor family pyrin domain containing 3 (NLRP3), caspase‐1 and gasdermin (GSDM) and further promoting pyroptosis in cardiomyocytes [81]. Likewise, exosomes derived from the blood of patients with sepsis can promote apoptosis in myocardial cells via the hsa‐miR‐1262/solute carrier family 2 member 1 (SLC2A1) axis. Exosomal hsa‐miR‐1262 is elevated to downregulate the expression of SLC2A1, a key mediator in energy metabolism, leading to reduced aerobic glycolysis and increased apoptosis in cardiomyocytes [82]. Hence, exosomes derived from circulating blood during sepsis induce cardiomyopathy by triggering apoptosis and pyroptosis in cardiomyocytes and endothelial dysfunction.

### 4.2 Cardiomyocytes

The pathogenesis of SICM involves complex interactions between inflammatory responses, oxidative stress and mitochondrial dysfunction, all of which contribute to cardiomyocyte death, a central factor in the disease [96, 97]. Under stressed conditions, exosomes derived from cardiomyocytes affect the development of SICM through various molecular mechanisms. In the cardiac environment, exosomes released by cardiomyocytes are believed to initiate functional events in target cells by inducing an array of metabolism‐related processes [98]. Under conditions of glucose deprivation, cardiomyocyte‐derived exosomes are loaded with a broad repertoire of miRNA and proteins, which are taken in by endothelial cells and promote the transcription of pro‐angiogenic genes, thereby facilitating proliferation and angiogenesis [83]. These findings suggest cardiomyocyte‐derived exosomes exert a protective function through the induction of local neovascularisation during acute cardiac injury. Besides, exosomal lncRNA KLF3‐AS1 derived from cardiomyocytes exposed to ischemia/reperfusion (I/R) injury can be delivered into MSCs, in which KLF3‐AS1 promotes insulin‐like growth factor 1 (IGF‐1) production via the miR‐23c/STAT5B axis, thus enhancing cardiomyocyte viability and alleviating myocardial injury [84]. IGF‐1 is closely associated with the development and growth of cardiomyocytes by promoting cell growth and resisting cell death [99]. As a pyrogen, LPS derived from Gram‐negative bacterial infections causes cardiac tissue death by triggering inflammation and endothelial cell injury during SICM [100]. Intriguingly, following the treatment of cardiomyocytes with LPS, the size and quantity of exosomes are decreased, along with the reduced expression of exosomal proteins that are related to exosomal biogenesis, which is associated with increased cardiac cell death [11]. These results indicate that cardiomyocyte‐derived exosomes play a dual role in SICM, where they either exacerbate or alleviate myocardial injury.

### 4.3 Endothelial Cells

Endothelial cell dysfunction contributes to sepsis‐induced mortality and organ dysfunction. During sepsis, endothelial cells shift toward a pro‐apoptotic and pro‐inflammatory phenotype and are involved in the impairment of microcirculatory blood flow and cardiac injury [101, 102]. Exosomes derived from endothelial cells have been shown to influence cardiac function and remodelling during the progression of SICM. Heat shock protein A12B (HSPA12B) in endothelial cells is confirmed to protect against sepsis‐induced cardiac dysfunction by upregulating the expression of miR‐126, which reduces immune cell infiltration in the myocardium and mitigates cardiac injury via suppressing the expression of adhesion molecules [103]. Of interest, exosomal HSPA12B derived from endothelial cells also exerts a protective function in SICM. Compared with septic mice, HSPA12B^−/−^septic mice have higher serum levels of TNF‐α and IL‐1β, greater infiltrated macrophages in the myocardium and worsened cardiac dysfunction, implying the protective role of HSPA12B. When exosomal HSPA12B from endothelial cells is uptaken by LPS‐stimulated macrophages, HSPA12B reduces the production of TNF‐α and IL‐1β but increases the IL‐10 levels via downregulating NF‐κB activation and nuclear translocation, thus decreasing macrophage‐mediated pro‐inflammatory responses and sepsis‐induced cardiac injury and mortality [85]. Additionally, LPS binding to endothelial cells elicits endothelial activation and damage, manifested by the expression of pro‐inflammatory cytokines and adhesion molecules that contribute to sepsis [104]. LPS‐mediated endothelial dysfunction exaggerates inflammation, coagulopathy and vascular leakage, which increases the morbidity and mortality of patients with SICM [105]. LPS‐stimulated endothelial‐derived exosomes contain miRNAs that are crucial for cardiomyocyte protection. These miRNAs downregulate apoptosis‐related proteins such as BAK1, P53 and PTEN, thereby promoting cell survival and reducing myocardial injury in the context of sepsis [12]. Consistently, in LPS‐induced endothelial injury, anisodamine ameliorated inflammation, cardiomyocyte dysfunction and myocardial injury through exosome‐mediated regulation of the PI3K/AKT and NF‐κB signalling pathways [86]. Accordingly, endothelial‐derived exosomes show promise in protecting cardiomyocytes during sepsis.

### 4.4 Macrophages

Macrophages, including M1 and M2 phenotypes, play a crucial role in the pathophysiology of SICM. M1 macrophages are known for their pro‐inflammatory role, which can lead to increased myocardial inflammation and tissue damage by producing inflammatory cytokines such as TNF‐α and IL‐1β [106]. M2 macrophages play a protective role in SICM by promoting anti‐inflammatory responses and tissue repair. They secrete anti‐inflammatory cytokines like IL‐10, which help mitigate myocardial damage [107]. The balance between these macrophage phenotypes is critical in determining the progression and resolution of SICM [108]. Exosomes derived from various macrophage phenotypes influence cardiomyocyte apoptosis, mitochondrial function and inflammatory responses. Pre‐treatment with GW4869, an inhibitor of exosome biogenesis, represses the release of pro‐inflammatory cytokines, such as TNF‐α, IL‐1β and IL‐6 in LPS‐stimulated macrophages, and reduces cardiac inflammation and myocardial depression in septic mice, suggesting the pro‐inflammatory role of macrophage‐derived exosomes [13]. The interaction between thioredoxin‐interacting protein (TXNIP) and NLRP3 is crucial for inflammasome activation and participates in inflammatory responses associated with sepsis [109]. In mice with sepsis‐induced myocardial dysfunction, oxidative stress increases the level of exosomal TXNIP‐NLRP3 complex derived from monocytes, which can be transported into the resident heart macrophages, where it activates caspase‐1 and cleaves inactive IL‐1β and IL‐18, thereby exacerbating cardiovascular inflammation [87]. Likewise, it is found that exosomal lncRNA Snhg14 derived from M1 macrophages can be delivered into cardiomyocytes, which promotes the release of pro‐inflammatory cytokines, myocardial apoptosis and oxidative stress in LPS‐treated cardiomyocytes, as well as mitigates cardiac damage in septic mice by suppressing miR‐181a‐5p and activating the HMGB1/NF‐κB signalling pathway [88]. Thus, these findings indicate that exosomes from macrophages accelerate SICM progression. However, macrophage‐derived exosomes also exert a protective function in SICM. For instance, it has been shown that exosomes from IL‐1β stimulated macrophages are enriched with miR‐146a, which reduces myocardial injury by inhibiting inflammatory responses and improving mitochondrial function through the MAPK4/DRP1 signalling pathway, accompanied by decreased serum myocardial enzymes and oxidative stress, thereby relieving myocardial injury during sepsis [89]. Coincidentally, M2 macrophage‐derived exosomal miR‐24‐3p displays cardioprotective effects on LPS‐induced septic mice by reducing cardiomyocyte apoptosis and improving cardiac function. Further mechanistic investigation revealed that miR‐24‐3p can inhibit the expression of tumour necrosis factor superfamily member 10 (TNFSF10), which is correlated with immune unresponsiveness to secondary heterologous bacterial infection after sepsis [90].

### 4.5 Platelet

Platelets are not only involved in coagulation and thrombosis but also in inflammatory processes that exacerbate myocardial depression. Platelet activation in sepsis is driven by interactions with pathogens, leading to the formation of microthrombi that cause ischemic damage in the heart and thus cardiac dysfunction [110]. Exosomes released by platelets in septic patients have been identified as contributors to myocardial depression. These exosomes produce superoxide and induce apoptosis in vascular cells, which may lead to inotropic dysfunction in the heart [91]. During sepsis, exosomes are overproduced in platelets exposed to LPS, and they trigger caspase‐3 activation and apoptosis in endothelial cells by upregulating the expression of superoxide, NO and peroxynitrite, thus contributing to septic vascular dysfunction [92]. Besides, platelets are verified to be potent activators of neutrophil extracellular trap (NET) formation during sepsis [111]. It is reported that septic patient‐derived exosomes can increase the NET components, such as double‐stranded DNA and MPO‐DNA complexes, which are associated with disease severity. In septic mice, platelet depletion reduces plasma exosome concentration and NET formation, indicating that platelet‐derived exosomes promote the development of SICM. Mechanistic studies demonstrated that exosomal molecules, including high‐mobility group protein 1 (HMGB1), miR‐15b‐5p and miR‐378a‐3p, are responsible for the NET formation through the activation of the AKT/mTOR signalling pathway [93].

### 4.6 Other Cells

Cardiac fibroblasts, the most abundant cell type in the heart's interstitium, are crucial for maintaining the structural integrity of the myocardium. Under conditions of stress, such as sepsis, these cells undergo a transformation into myofibroblasts and secrete exosomes that contain pro‐inflammatory cytokines, growth factors and matrix proteins [22]. Exosomes from cardiac fibroblasts carry pro‐inflammatory molecules, such as TNF‐α and IL‐1β, which contribute to the systemic inflammatory response, thus exacerbating myocardial injury and worsening cardiac dysfunction [112]. These exosomes also influence the deposition and turnover of extracellular matrix proteins, such as collagen and fibronectin, contributing to myocardial fibrosis [113]. Recent studies have identified miR‐23a‐3p in fibroblast‐derived exosomes that regulate fibrotic processes via the promotion of oxidative stress injury and ferroptosis, thereby enhancing fibrosis and cardiac remodelling [114].

Cardiac Progenitor Cells (CPCs), which are multipotent cells capable of differentiating into various cardiac cell types, including cardiomyocytes, endothelial cells and smooth muscle cells, also release exosomes with significant therapeutic potential in SICM [115]. CPC‐derived exosomes are rich in growth factors such as vascular endothelial growth factor and basic fibroblast growth factor, which stimulate angiogenesis and tissue repair [116]. Additionally, CPC‐derived exosomes carrying miR‐210, miR‐132 and miR‐146a‐3p have been shown to enhance tube formation in endothelial cells and protect cardiomyocytes from apoptosis, thus mitigating cardiac dysfunction [117]. The cardioprotective properties of exosomes from CPCs are further exemplified in their ability to reduce oxidative stress and improve mitochondrial function. By transferring antioxidant enzymes and proteins involved in cellular energy metabolism, these exosomes help restore cellular homeostasis and protect cardiac myocytes from ischemic damage and inflammation [118].

Exosomes from both cardiac fibroblasts and CPCs can modulate cardiac inflammation, fibrosis and remodelling, which are hallmark features of SICM. The delivery of CPC‐derived exosomes to the heart could promote myocardial repair, reduce inflammation and mitigate fibrosis, thereby offering a novel avenue for treating SICM. Moreover, exosomal miRNAs from these sources could be exploited as diagnostic or therapeutic biomarkers in SICM. Their potential to modulate key signalling pathways involved in inflammation, fibrosis and apoptosis makes them attractive targets for intervention.

## 5 Gender‐ and Age‐Dependent Differences in Exosome Biology and Their Cardioprotective Effects

Recent research indicates that the biological properties of exosomes, including their composition and functional activity, are influenced by both gender and age [119]. Understanding these differences is essential for optimising the therapeutic targeting of exosomes in SICM. Several studies have highlighted significant gender‐based differences in exosome secretion, composition and functional properties. For instance, female‐derived exosomes have been shown to exhibit a distinct protein and RNA profile compared to male‐derived exosomes, influencing their immunomodulatory and cardioprotective functions. One of the most notable gender‐based differences is related to the hormonal regulation of exosome production. Oestrogen, which is more abundant in females, has been implicated in the increased secretion of exosomes from cardiomyocytes, which may enhance their cardioprotective functions [120]. This effect is thought to be mediated through oestrogen receptors, which can modulate exosome cargo, including miRNAs involved in anti‐inflammatory and anti‐apoptotic pathways [121]. In contrast, male‐derived exosomes tend to exhibit a higher level of pro‐inflammatory cytokines and signalling molecules, which may exacerbate inflammation. For example, exosomes from male septic animals have been shown to carry higher levels of TNF‐α and IL‐6, which can potentiate myocardial dysfunction and impair cardiac recovery [122]. Thus, the differences in exosomal cargo between males and females could have implications for the progression of SICM and for the therapeutic targeting of exosomes as a strategy to mitigate cardiac injury.

Age is another critical factor that influences exosome biology, with elderly individuals exhibiting distinct alterations in exosome composition and function compared to younger individuals. During sepsis, aged individuals are vulnerable to cardiovascular dysfunction due to impaired cardiac repair mechanisms and heightened systemic inflammation. Exosomes derived from aged individuals show alterations in their protein and RNA cargo, including decreased levels of miRNAs with cardioprotective properties, such as miR‐21 and miR‐146a, which have been shown to regulate inflammation and fibrosis in cardiac tissue [123]. Interestingly, exosomes from aged mice are confirmed to have diminished cardioprotective effects during sepsis. These exosomes are less efficient in attenuating inflammation and protecting against cell injury when compared to exosomes derived from young animals [124]. This suggests that aging may lead to a decline in the quality and efficacy of exosomes as therapeutic agents in SICM. The underlying mechanisms are likely multifactorial, involving age‐related changes in exosome biogenesis, secretion and alterations in the molecular machinery responsible for packaging functional miRNAs and proteins. Additionally, the mitochondrial dysfunction that is often seen in aging contributes to altered exosome content. Mitochondrial‐derived damage‐associated molecular patterns present in exosomes from aged individuals may also promote a pro‐inflammatory environment, further impairing cardioprotective effects [125]. Therefore, age‐dependent modifications in exosome biology represent a critical barrier to their therapeutic efficacy in older adults, necessitating age‐specific strategies for exosome‐based therapies.

Given the profound influence of gender and age on exosome biology, a better understanding of these differences could enhance the therapeutic potential of exosome‐based interventions for SICM. Tailoring exosome‐based therapies to account for these factors is essential to maximise their cardioprotective effects. For example, enhancing the oestrogen‐dependent release of protective exosomes in females or modulating the composition of male‐derived exosomes to reduce pro‐inflammatory cargo could be promising approaches. Similarly, strategies aimed at rejuvenating exosome function in aging individuals, such as through the use of mitochondrial‐targeted therapies, could enhance their therapeutic potential in SICM [126].

## 6 Exosomes Derived From MSCS as Potential Therapeutic Targets in SICM

MSCs are recognised to exist in nearly all postnatal organs and tissues, such as bone marrow, adipose tissue, umbilical cord and placenta [127, 128]. Under suitable conditions, MSCs possess the capacity to differentiate into osteoblasts, adipocytes and chondroblasts, rendering them promising candidates for therapeutic interventions owing to their plastic properties [129]. MSC‐derived exosomes play a critical role in mediating the therapeutic effects of MSCs. Exosomes released by MSCs facilitate tissue repair and regeneration by transferring growth factors and miRNAs that enhance cell proliferation and differentiation [130]. In SICM, MSC‐derived exosomal miRNAs exert anti‐inflammatory effects to halt disease progression. For example, exosomal miR‐223 from MSCs is verified to inhibit cardiomyocyte apoptosis, inflammatory responses and cardiac dysfunction by downregulating the expression of semaphorin 3a (SEMA3A) and signal transducer and activator of transcription 3 (STAT3) in septic mice [131]. Also, miR‐146a‐5p within MSC‐derived exosomes can be delivered into LPS‐induced cardiomyocytes where it facilitates cell proliferation and represses apoptosis; moreover, it inhibits the inflammatory response of myocardial tissues of septic mice via reducing MYBL1 expression [132]. In addition, it is reported that miR‐412‐5p‐loaded exosomes of MSCs ameliorate LPS‐induced inflammation in cardiomyocytes by inhibiting the expression of inflammatory mediators, including NO, prostaglandin E2 (PGE2) and ROS and the secretion of pro‐inflammatory cytokines like IL‐1β and IL‐6, via inactivating the MAPK signalling pathway [17]. By inhibiting the miR‐497‐5p/MG53 axis in cardiomyocytes, circRNA RTN4 from MSC‐derived exosomes is verified to lessen the production of inflammatory factors, including ROS, IL‐1β, IL‐6 and TNF‐α, and increases the activity of superoxide dismutase (SOD) and glutathione (GSH), thereby alleviating myocardial apoptosis and cardiac injury in LPS‐treated cardiomyocytes and septic rats [133]. These studies highlight the significant potential of MSC‐derived exosomes in modulating cardiac inflammation and myocardial apoptosis in SICM. Intriguingly, MSC‐derived exosomes loaded with sweroside, biologically active natural iridoids with potent anti‐inflammatory and antioxidant activity, decrease the generation of pro‐inflammatory cytokines and oxidative stress in septic rats, along with suppressed cardiomyocyte apoptosis and increased myocardial survival, thus impeding sepsis‐induced myocardial injury [18].

Bone marrow mesenchymal stem cells (BMSCs) are multipotent stem cells derived from bone marrow and exhibit immunomodulatory capacity that render them appropriate for alleviating inflammatory and immune‐mediated conditions like sepsis‐induced myocardial injury [134]. It has been demonstrated that exosomes derived from LPS‐treated BMSCs can inhibit M1 polarisation and promote M2 polarisation by suppressing the NF‐κB but activating the AKT1/AKT2 signalling pathway. In vivo study further confirms that these exosomes alleviate myocardial inflammation and cardiomyocyte apoptosis in mice with myocardial infarction [135]. Additionally, exosomal miR‐181a‐5p from BMSCs exposed to LPS challenge reduces the expression of TNF‐α and IL‐1β and increases the levels of SOD1 and SOD2 in H_2_O_2_‐stimulated cardiomyocytes by downregulating activating transcription factor 2 (ATF2), which further attenuates myocardial inflammation and oxidative stress [136]. Similarly, exosomes derived from BMSCs, acting as carriers for delivering miR‐141 into myocardial tissues, reduce the production of creatine kinase MB and lactate dehydrogenase and inhibit inflammatory infiltration and cell apoptosis by suppressing the expression of PTEN and subsequently enhancing the activity of β‐catenin, thereby halting myocardial injury in septic mice [137]. Thus, BMSC‐derived exosomes protect against sepsis‐induced myocardial injury by modulating macrophage polarisation and cardiomyocyte death.

MSCs obtained from umbilical cord tissue are valued for their high proliferative capacity and immunomodulatory properties, positioning them as promising candidates for regenerative and therapeutic interventions in sepsis‐induced myocardial injury [138, 139]. Exosomes derived from human umbilical cord MSCs modulate inflammatory responses and myocardial injury, which are involved in inflammation resolution and tissue repair in SICM. For instance, exosomes from these MSCs can facilitate fibroblast‐to‐myofibroblast differentiation in cardiac inflammatory environments, alleviating inflammatory responses and cardiomyocyte apoptosis [140]. The cardiac fibroblast‐to‐myofibroblast differentiation leads to cardiac remodelling, characterised by an increase in collagen, fibronectin and elastin, which helps to form a fibrous scar tissue that replaces the damaged myocardial tissue, providing structural support to the heart [141]. Consistently, umbilical cord MSC‐derived exosomal PTEN‐induced putative kinase 1 (PINK1) mRNA is transferred to recipient cardiomyocytes to upregulate PINK1 expression, which promotes mitochondrial calcium efflux and relieves mitochondrial calcium overload and myocardial injury by activating the PKA/NCLX signalling pathway [142].

**FIGURE 3:**
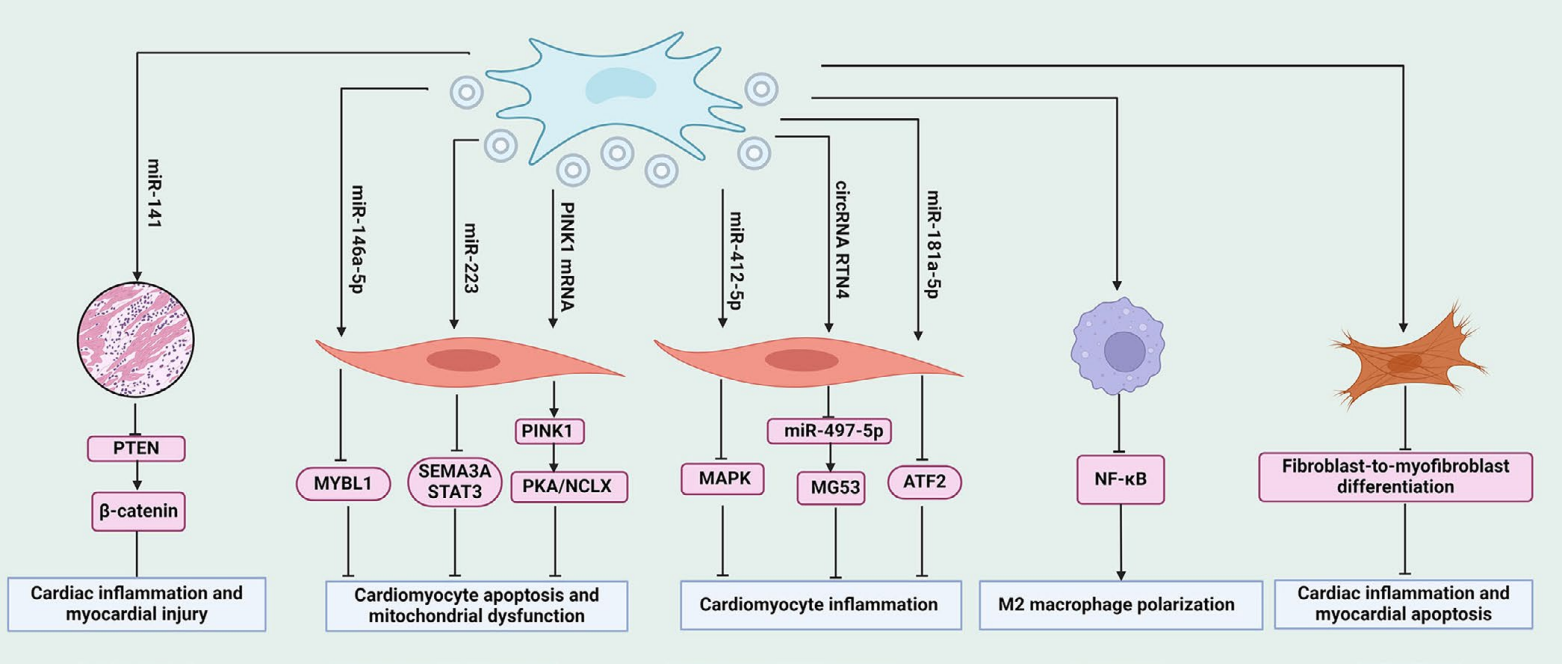
The role of MSC‐derived exosomes in sepsis‐induced cardiomyopathy. MSC‐derived exosomal components, such as mRNA and non‐coding RNAs, exert crucial roles in alleviating cardiac inflammation, cardiomyocyte apoptosis, macrophage polarisation, and myocardial injury via acting on various cell types, such as cardiomyocytes, macrophages, and myofibroblasts, thereby affecting the functions of recipient cells through regulating diverse signalling pathways like NF‐κB, PTEN/β‐catenin and MAPK. ATF2, activating transcription factor 2; circRNA, circular RNA; lncRNA, long non‐coding RNA; MAPK, mitogen‐activated protein kinase; MSCs, mesenchymal stem cells; miRNA, microRNA; PINK1, PTEN‐induced putative kinase 1; SEMA3A, semaphorin 3a; STAT3, signal transducer and activator of transcription 3. ⊥ indicates an inhibitory effect and → indicates a promoting effect.

In conclusion, MSC‐derived exosomes can alleviate SICM progression via modulating inflammatory responses and myocardial injury (Figure 3; Table 2). In this regard, MSC‐derived exosomes have promising potential in SICM treatment. Additionally, engineering exosomes from MSC to deliver therapeutic components to the heart represents a promising avenue for targeted therapy, but effective delivery of therapeutic exosomes is critical for their clinical success. Several delivery methods are being explored to ensure that exosomes reach their target cells in sufficient quantities while maintaining their stability and functionality [143]. One of the main challenges in developing exosome‐related therapies is ensuring efficient delivery to target cells. Future research should focus on optimising delivery systems, such as using nanoparticles or liposomes to enhance the stability and bioavailability of exosomal components.

**TABLE 2:** The therapeutic effects of MSC‐derived exosomes in sepsis‐induced cardiomyopathy in vivo.

Number of exosomes	Time and route of administration	Targets	Animal model	Animal gender and strain	Ref.
1.5 mg extracted exosomes from MSCs	Immediate injection after lipopolysaccharide treatment, intraperitoneal injection	Unreported	Lipopolysaccharide‐induced myocardial injury	Male C57/B6J mice	[18]
150 μL culture medium from MSCs	1 h post‐CLP, tail vein injection	SEMA3A and STAT3	CLP‐induced septic mice	Male and female C57BL/6 mice	[131]
Unreported	Unreported	MYBL1	CLP‐induced septic mice	C57BL/6 mice	[132]
100 μL culture medium from MSCs	3 consecutive days after CLP, intraperitoneal injection	miR‐497‐5p/MG53	CLP‐induced septic rats	Wistar rats	[133]
Unreported	Immediate injection after LAD ligation, myocardium injection	AKT1/AKT2	LAD ligation‐induced myocardial infarction model	Male C57BL/6 mice	[135]
2 μg exosome/g weight	Immediate injection after CLP, tail vein injection	PTEN/β‐catenin	CLP‐induced septic mice	Male KM mice	[137]
400 μg exosome from MSCs	Immediate injection after LAD ligation, intramyocardial injection	Unreported	LAD ligation‐induced myocardial infarction model	Male Sprague–Dawley rats	[140]
2 μg exosome/g weight	0 h and 6 h after CLP, intraperitoneal injection	PKA/NCLX	CLP‐induced septic mice	Male and female C57BL/6 mice	[142]

## 7 Targeting Exosomes by Drugs and Natural Components for SICM Treatment

A growing body of studies suggests that various drugs and bioactive natural compounds can influence the secretion and internalisation of exosomes with cardioprotective properties. For example, ticagrelor, a P2Y12 receptor antagonist, has been shown to enhance the release of cardioprotective exosomes from CPCs. It attenuates hypoxia‐induced cell apoptosis through acute phosphorylation of ERK42/44 [144]. Ticagrelor‐pretreated cardiomyocyte‐derived EVs decrease aberrant ROS production, prevent the development of apoptosis and ER stress and alleviate oxidative stress. EVs derived from ticagrelor‐pretreated cardiomyocyte cells enhance endothelial cell migration and tube formation [145]. Besides, ticagrelor attenuates the release of prothrombotic EV concentrations in plasma after acute myocardial infarction, thereby contributing to improved cardiac function [146]. Simvastatin, a potent statin, is verified to upregulate decorin and downregulate periostin in cardiomyocyte‐derived exosomes, thus protecting against cardiac fibrosis [147]. Moreover, natural compounds like sulforaphane, polyphenols, omega‐3 fatty acids and flavonoids have been reported to enhance exosome release from fibroblasts with high tropism for cardiomyocytes, further supporting their therapeutic potential in sepsis‐induced cardiac injury. For instance, norepinephrine promotes the release of fibroblast‐derived EVs, which further stimulate the proliferation of vascular smooth muscle cells and excessive sympathetic activation‐related vascular remodelling [148]. Sulforaphane, an edible compound, enhances the release of fibroblast‐derived cardioprotective exosomes with tropism towards cardiomyocytes, which reduce oxidative stress, hypertrophy and scar size and improve contractility, thereby preventing the onset of heart failure [149]. These agents influence the exosomal cargo, including miRNAs, proteins and lipids, which can modulate cellular processes such as apoptosis, inflammation and oxidative stress, thus enhancing cardioprotection.

The pharmacological and natural modulation of exosome release and uptake represents a novel therapeutic strategy in the treatment of SICM. By targeting the biogenesis, release and uptake of exosomes, it is possible to reduce myocardial inflammation, fibrosis and injury during SICM. The development of exosome‐based therapies, either as drug delivery systems or as modulators of endogenous exosome biology, holds great promise for future clinical applications. However, challenges remain in translating these preclinical findings into clinical practice, including the need for improved targeting and delivery systems for exosome‐based therapies. Additionally, understanding the long‐term effects of exosome modulation on cardiac remodelling and function is critical to the success of these therapeutic strategies.

## 8 Conclusion

This review highlights the multifaceted roles of exosomes derived from circulating blood, cardiomyocytes, endothelial cells, macrophages and platelets, in modulating inflammatory responses, oxidative stress and cardiomyocyte apoptosis during SICM progression. Exosomes carry a broad spectrum of bioactive molecules, including proteins, lipids, mRNA and miRNAs, which facilitate the transmission of signals among these cell types, influencing the progression of sepsis and its associated cardiac dysfunction. MSC‐derived exosomes loaded with specific components like miRNAs could offer targeted therapies, reducing inflammatory responses and promoting cardiac repair. While exosome‐based therapies hold promise for treating SICM, several challenges remain. These include optimising the isolation and production of exosomes on a large scale, ensuring the stability and efficacy of exosomal cargo and addressing potential off‐target effects. While exosomes from various cell types appear to harbour opposing effects in SICM, the mechanisms that govern their release, uptake and function within the heart are not yet fully elucidated. Future research should focus on a deeper understanding of the molecular mechanisms by which exosomes affect the pathophysiology of SICM, such as inflammatory responses, myocardial contractile impairment and endothelial dysfunction. Furthermore, the potential therapeutic applications of exosomes derived from MSCs as vehicles for targeted drug delivery are still in their infancy and require more rigorous validation in clinical settings. Importantly, the safety and efficacy of exosome‐based therapies must be thoroughly evaluated, including assessing potential immune reactions and long‐term outcomes. Thus, it is imperative to bridge the existing knowledge gap between preclinical models and human clinical data. The incorporation of clinical and human‐based studies into future research efforts will be crucial in establishing the true translational potential of exosome‐based therapies for SICM. Prospective studies in sepsis patients are needed to identify exosomal biomarkers that could serve as diagnostic or prognostic tools in SICM. Additionally, longitudinal studies that track exosome levels in sepsis patients could offer critical insights into their role in disease progression and recovery. Clinical trials evaluating the therapeutic efficacy of exosome‐based interventions, such as exosome‐mediated drug delivery and exosomal microRNA targeting, are essential to determine whether these approaches can be translated into effective treatments for SICM.

## Author Contributions

**Rui Fan:** writing – original draft (equal), writing – review and editing (equal). **Han Liu:** writing – original draft (equal), writing – review and editing (equal). **Qun Liang:** conceptualization (lead), project administration (lead).

## Conflicts of Interest

The authors declare no conflicts of interest.

## Data Availability

This paper is exempt from data sharing.
